# In Vitro and In Vivo Evaluation of Lactoferrin-Modified Liposomal Etomidate with Enhanced Brain-Targeting Effect for General Anesthesia

**DOI:** 10.3390/pharmaceutics16060805

**Published:** 2024-06-14

**Authors:** Ailing Wu, Houyin Shi, Luhan Yang, Hao Zhang, Xichen Nan, Dan Zhang, Zhuo Zhang, Chun Zhang, Siwei Chen, Xiujuan Fu, Lilan Ou, Lulu Wang, Yanyan Shi, Hao Liu

**Affiliations:** 1 Department of Anesthesiology, The First People’s Hospital of Neijiang, Neijiang 641100, China; 2 Department of Orthopedics, The Affiliated Traditional Chinese Medicine Hospital, Southwest Medical University, Luzhou 646699, China; 3 School of Pharmacy, Southwest Medical University, No.1 Section 1, Xiang Lin Road, Longmatan District, Luzhou 646699, China

**Keywords:** etomidate, brain targeting, lactoferrin, liposome, anesthesia, toxicity

## Abstract

Etomidate is a general anesthetic that has shown good hemodynamic stability without significant cardiovascular or respiratory depression. Despite several kinds of dosage forms having been reported for this drug, formulation types are very limited in clinical practice, and brain-targeted formulations for this central nervous system (CNS) drug have been rarely reported. Moreover, studies on the biocompatibility, toxicity, and anesthetic effects of the etomidate preparations in vivo were inadequate. The present study was to develop lactoferrin-modified liposomal etomidate (Eto-lip-LF) for enhanced drug distribution in the brain and improved anesthetic effects. Eto-lip-LF had good stability for storage and hemocompatibility for intravenous injection. Compared with the non-lactoferrin-containing liposomes, the lactoferrin-modified liposomes had notably enhanced brain-targeting ability in vivo, which was probably realized by the binding of transferrin with the transferrin and lactoferrin receptors highly distributed in the brain. Eto-lip-LF had a therapeutic index of about 25.3, higher than that of many other general anesthetics. Moreover, compared with the commercial etomidate emulsion, Eto-lip-LF could better achieve rapid onset of general anesthesia and rapid recovery from anesthesia, probably due to the enhanced drug delivery to the brain. The above results demonstrated the potential of this lactoferrin-modified liposomal etomidate to become an alternative preparation for clinical general anesthesia.

## 1. Introduction

**Figure 1:**
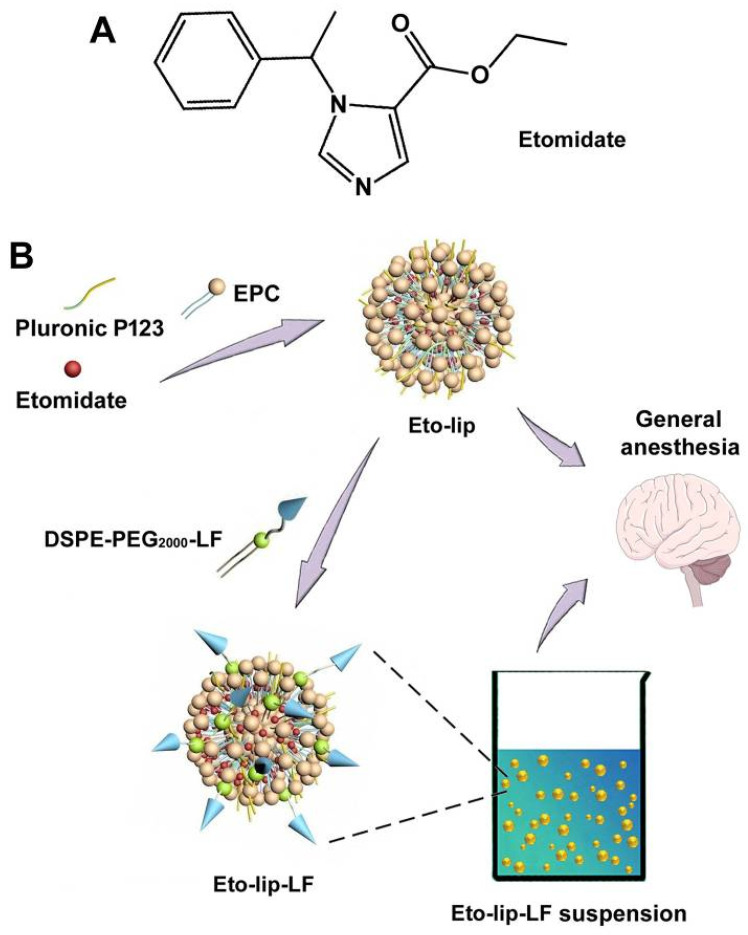
(**A**) The chemical structure of etomidate; (**B**) Schematic representation for the composition and structure of lactoferrin-modified liposomal etomidate (Eto-lip-LF) and non-lactoferrin-containing liposomal etomidate (Eto-lip).

Etomidate is a first-line general anesthetic that can induce anesthesia via positive allosteric modulation of the γ-aminobutyric acid type A receptor (Figure 1A) [1]. At the clinical doses for general anesthesia, etomidate showed good hemodynamic stability without significant cardiovascular or respiratory depression. Due to the low water solubility of this imidazole derivative, etomidate has been made into injectable preparations with the help of solubilizers or co-solvents [2,3]. Geng et al. conducted optimization experiments to achieve the optimal formula for etomidate emulsion [3]. Their optimized etomidate emulsion prepared using soybean oil, medium-chain triglycerides, egg lecithin, poloxamer 188, sodium oleate, and glycerol showed good stability for long-term storage and safety for injection. In another study, Liu et al. prepared etomidate-encapsulated nanoparticles using self-assembling peptides composed of glycine, glutamine, and tyrosine [4]. Their study suggested that the peptide nanoparticles had excellent drug-loading capacity and biocompatibility. Our team prepared an etomidate-loaded micelle based on Pluronic F108 and soybean oil as a solubilizer [5]. The results suggested that the micelle could well maintain the drug efficacy of etomidate in rats and had the potential to develop as a short-acting anesthesia preparation.

Despite several kinds of dosage forms having been reported for etomidate, for the moment formulation types are very limited in clinical practice, and brain-targeted formulations for this CNS drug have been rarely reported. Free distribution of etomidate in the body could probably slow down the onset of anesthesia and recovery from anesthesia. However, for general anesthetics, rapid onset of anesthesia and recovery from anesthesia are essential for better anesthetic effects and clinical safety [6]. Also, in vivo studies on the biocompatibility, toxicity, and anesthetic effects of etomidate preparations were still few. It should be noted that the addition of some organic solvents and co-solvents in the etomidate preparations may bring about health-related issues that should not be neglected. For instance, propylene glycol has been used as a solubilizer in etomidate aqueous injections, but this substance has been reported to lead to a variety of adverse effects such as hyperosmolarity, cardiac arrhythmia, and lactic acidosis [2,7,8]. The considerable amount of soybean oil as the oil phase in the etomidate emulsions may cause problems for patients with obesity or hyperlipemia and, as a nutrient, could also add to the risk of microbial contamination in the formulation. The above facts made it rational to develop a brain-targeted formulation for etomidate with an acceptable drug concentration for injection and good safety.

The application of hybrid or composite nanocarriers made from different materials has become a useful strategy to achieve multiple purposes simultaneously [9,10,11,12,13,14]. We previously prepared a mixed micelle for etomidate using different Pluronic polymers [8]. The mixed micelle made use of the advantages of different materials, resulting in better properties for etomidate encapsulation without the incorporation of other organic solvents. However, the biodistribution and biocompatibility of this mixed nanocarrier in vivo needed further investigation, and its stability and drug-encapsulating ability needed to be strengthened. It is noteworthy that some biomaterials functionalized with permeation enhancers for the blood–brain barrier (BBB) or specific ligands (e.g., transferrin and lactoferrin) can be introduced into a nanocarrier to facilitate drug accumulation in the brain, thus enhancing the drug efficacy and reducing the adverse effects of etomidate, such as adrenal toxicity and myoclonus [15].

During the past decades, liposomes have been widely tested for drug delivery to the brain [16,17,18,19,20]. A number of pharmaceutical studies have demonstrated the many advantages of liposomes, including good biocompatibility, biodegradability, flexibility in size and surface charge, and versatility for various applications, making them an advanced nanocarrier system for targeted drug delivery [21,22,23]. Recently, Zhou et al. developed a biomimetic nano-drug with a paclitaxel-loaded liposome as the core and a hybrid cell membrane as the envelope [24]. This nano-drug could effectively go across the BBB, target brain tumor lesions, and significantly inhibit the growth of gliomas. Wang et al. engineered a hybrid nanovesicle based on a blood exosome and a tLyp-1 peptide-modified liposome [25]. With the addition of the transferrin receptor, the hybrid nanovesicle co-loaded with salvianolic acid B and cryptotanshinone exhibited increased BBB transcytosis into the brain parenchyma and synergistic drug effects on glioma. Moreover, liposomes have also been administered via the intranasal route to grant more direct access to the brain [26,27,28]. These results suggested that the development of hybrid or composite liposomes could provide good strategies for brain-targeted drug delivery and enhanced drug efficacy. Nevertheless, some issues with liposomes, such as the composition of aggregates, should not be neglected when they are used for drug delivery [29,30].

Pluronic P123 is an amphiphilic triblock copolymer consisting of poly(ethylene glycol) (PEG) and poly(propylene glycol) (PPG). Due to its low hydrophile–lipophile balance (HLB) value (i.e., about 8), Pluronic P123 can be considered for the delivery of drugs with significant lipophilicity [31]. In addition, Pluronic P123-based nanocarriers have shown the potential to inhibit the P-glycoprotein on the blood–brain barrier (BBB) [32]. Liu et al. developed tryptophan derivate-functionalized lamotrigine-loaded mixed micelles (TD-PF/LTG) using Pluronic P123 and Pluronic F127 for epilepsy therapy [33]. In their animal study, TD-PF/LTG presented a significantly increased brain/plasma ratio of the drug levels in the P-gp overexpressed rat model, indicating excellent targeting efficiency. Wang et al. prepared chitosan-functionalized myricetin-loaded mixed micelles using Pluronic P123 and Pluronic F68 (MYR-MCs) for glioblastoma treatment [34]. It was found that MYR-MCs could efficiently transport the drug across the BBB and significantly increase the accumulation and retention time of myricetin in the brain. These studies demonstrated that Pluronic P123 could be a promising loading material to facilitate the delivery of etomidate to the brain.

Lactoferrin is a biologically safe protein that can bind to transferrin, and lactoferrin receptors are highly distributed in brain endothelial cells [35]. This property has made it an excellent ligand for nanocarriers to enhance their accumulation in the brain. Many kinds of lactoferrin-modified nanoformulations have been investigated as candidate preparations for Alzheimer’s disease, Parkinson’s disease, and brain tumors and have exhibited considerable efficiency in delivering the drug in the CNS [36]. Based on the above results, it was hypothesized that the incorporation of lactoferrin together with Pluronic P123 in the liposome could achieve notably enhanced drug distribution in the brain.

In this study, we developed lactoferrin-modified Pluronic P123-incorporated liposomal etomidate (Eto-lip-LF) as a novel general anesthetic nanoformulation with enhanced drug distribution in the brain and improved general anesthetic effects (Figure 1B). The size distribution, zeta potential, morphology, and drug loading efficiency of Eto-lip-LF were studied for characterization. The stability, drug release profile, and blood compatibility of Eto-lip-LF were investigated via in vitro experiments. The in vivo brain-targeting ability of the lactoferrin-modified liposomes was studied based on fluorescence imaging. Finally, the in vivo toxicity and anesthetic effects of Eto-lip-LF were evaluated in rats.

## 2. Materials and Methods

### 2.1. Materials

Etomidate (>98%), DiR (1,1′-dioctadecyl-3,3,3′,3′-tetramethylindotricarbocyanine iodide), Pluronic P123, and egg L-α-phosphatidylcholine (EPC) were supplied by Shanghai Aladdin Biochemical Technology Co., Ltd. (Shanghai, China). The commercial etomidate emulsion was supplied by Jiangsu Nhwa Pharmaceutical Co., Ltd. (Xuzhou, China). Lactoferrin-modified PEGylated 1,2-distearoyl-*sn*-glycero-3-phosphoethanolamine (DSPE-PEG_2000_-LF) was supplied by Luzhou Kejin Biological Products Co., Ltd. (Luzhou, China). All other chemicals and solvents were of analytical grade.

### 2.2. Animals

Male Sprague–Dawley (SD) rats (250–300 g) and male New Zealand rabbits (2.5–2.8 kg) were provided by Chongqing Tengxin Bier Experimental Animal Sales Co., Ltd. (Chongqing, China). The animals were fed under controlled pathogen-free conditions and acclimated to the new housing conditions for over two weeks before experiments.

### 2.3. Preparation of Eto-Lip-LF

The lactoferrin-modified liposomal etomidate (Eto-lip-LF) was produced based on a thin-film hydration method [9]. Etomidate (10 mg), EPC (85 mg), Pluronic P123 (15 mg), and DSPE-PEG_2000_-LF (60 mg) were dissolved in 5 mL dichloromethane. Then, the solution formed a thin film in a RE-2000B rotary evaporator (Yarong Instruments, Shanghai, China). Finally, Eto-lip-LF was obtained by adding 5 mL phosphate-buffered solution (PBS, pH 7.4) to hydrate the film and homogenized using a JY96-IIN probe sonicator (Huxi Industrial, Shanghai, China). The non-lactoferrin-containing liposomal etomidate (Eto-lip) was prepared similarly without DSPE-PEG_2000_-LF. Also, DiR-encapsulated liposomes (i.e., DiR-lip-LF and DiR-lip) with a dye concentration of 50 nmol/mL were obtained utilizing DiR instead of the drug.

### 2.4. Characterizations and Stability Study

The etomidate concentrations of Eto-lip-LF and Eto-lip were measured using a UV-8000S UV–Vis spectrophotometer (Shanghai Metash Instruments, Shanghai, China) at 243 nm wavelength, according to the previously reported method [37]. The particle size distribution, polydispersity index (PDI), and zeta potential were measured on a Nano ZS Zetasizer (Malvern Instruments, Malvern, UK). The measurement of each of the above four parameters was conducted in triplicate. Transmission electron microscopy (TEM) was used to study the morphology of Eto-lip-LF and Eto-lip, for which the liposome suspension was negative-stained with phosphotungstic acid before visualization using an H-600 transmission electron microscope (Hitachi, Tokyo, Japan).

To study the drug loading efficiency (DLE) and drug loading capacity (DLC), the liposome suspensions of Eto-lip-LF and Eto-lip were filtered by centrifugation using Amicon ultra centrifugal filters (MWCO 3000 Da, Merck KGaA, Darmstadt, Germany) [38]. The concentration of free etomidate was measured using spectrophotometry as described above. Then, the DLE and the DLC were calculated as follows: DLE = (the weight of loaded etomidate ÷ the total weight of input etomidate) × 100%; DLC = (the weight of loaded etomidate ÷ the total weight of the liposomal etomidate) × 100%. The measurements of DLE and DLC were conducted in triplicate, respectively.

The stability of Eto-lip-LF and Eto-lip was studied in terms of drug concentration, particle size distribution, PDI, and zeta potential. After production, the liposome suspensions were stored at room temperature (20–26 °C) and were investigated at different time points.

### 2.5. In Vitro Drug Release

The drug release of Eto-lip-LF and Eto-lip in vitro was investigated based on a dialysis method based on a previous study [39]. Briefly, the liposome suspension (2 mg/mL, 5 mL) or the commercial etomidate emulsion (2 mg/mL, 5 mL) was added to 20 mL PBS (pH 7.4). For the control group, 10 mg etomidate was dissolved in 25 mL PBS. Each sample was placed in a dialysis membrane (MWCO 2500 Da). Then, 400 mL of fresh PBS was added as the external phase. During the dialysis experiments, the temperature was kept at about 37 °C, and the released etomidate in the external PBS was investigated using spectrophotometry as described above. The drug-release experiment was conducted in triplicate for each formulation.

### 2.6. Hemolytic Activity

The hemolytic activity of the liposomal etomidate was tested using red blood cells (RBCs) from New Zealand rabbits based on the methods of some previous studies [40,41]. The RBCs separated from the blood of rabbits by centrifugation (1000 rpm, 10 min) were suspended in normal saline to obtain a 2% (*w*/*v*) cell suspension. Eto-lip-LF, or Eto-lip, was diluted in normal saline to make liposome suspensions of various etomidate concentrations. Then, 2 mL cell suspension was mixed gently with 2 mL pure water (positive control), normal saline (negative control), or different liposome suspensions (reaching the final concentrations of 0.05–0.25 mg etomidate/mL) and was incubated at 37 °C for 2 h, followed by centrifugation at 1000 rpm for 5 min. The absorbance (Abs) of the supernatant was detected at a wavelength of 540 nm. The hemolysis rate was calculated as follows: Hemolysis rate = (Abs from liposome suspension − Abs from negative control) ÷ (Abs from positive control − Abs from negative control) × 100%. The hemolysis rate of <5% indicated good hemocompatibility. The hemolytic activity experiment was conducted in triplicate for each formulation.

### 2.7. Fluorescence Imaging

The brain-targeting ability of the liposomes was studied using a fluorescence imaging technique, as reported in a previous study [14]. A free DiR solution with a dye concentration of 50 nmol/mL was prepared using ethanol/PBS (1:4, *v*/*v*) mixture as the solvent. Rats were randomized into three groups (n = 3). Each rat was injected with 300 μL of free DiR solution, DiR-lip, or DiR-lip-LF and sacrificed 2 h after the injection. Then, the brain tissue was removed and homogenized. For each rat, 200 μL of the homogenized tissue was added into a well of a 96-well plate. The fluorescence intensity of each well was determined using an In Vivo MS FX PRO imaging system (Carestream Health, Rochester, NY, USA), with a 750 nm wavelength for excitation and 830 nm for emission.

### 2.8. Acute Toxicity

The acute toxicity of Eto-lip-LF and Eto-lip was investigated using Dixon’s ‘up-and-down’ method, as reported in some previous studies [8,42,43]. Briefly, a rat was intravenously injected with the liposomal etomidate at a predetermined dose. The next rat received an elevated dose (by a 0.1 log interval) if the former rat survived; otherwise, it received a reduced dose (by a 0.1 log interval). For each experiment, when there were six evaluable samples, the median lethal dose (LD_50_) was calculated based on the order of the events (i.e., survival or death of the rats) and the tested dose values corresponding to these events, following the instructions of this method.

### 2.9. Maximum Tolerated Dose (MTD)

The MTD of Eto-lip-LF and Eto-lip for a single injection was investigated based on a reported observation method as described previously [44]. Briefly, rats were randomized into eight groups (n = 8). Each rat was intravenously injected with Eto-lip-LF or Eto-lip at an etomidate dose of 12, 14, 16, or 18 mg/kg, after which the body weight and the status of the rat were monitored for 10 days. The MTD was determined as the maximal dose that did not cause death, notable changes in the general appearance of the rats, or body weight loss over 15%.

### 2.10. In Vivo Anesthetic Effects

The anesthetic effects of the liposomal etomidate in vivo were evaluated in terms of the onset time and the action duration, which were determined based on the loss of righting reflex (LORR) in rats, following the methods as reported previously [5,45]. LORR meant the failure (due to anesthesia) of the rat to turn all its paws towards the ground twice from its supine position.

To determine the proper dose for the induction of anesthesia, the median effective dose (ED_50_) for etomidate-induced LORR in rats was investigated using the ‘up-and-down’ method as described above. A rat was intravenously injected with Eto-lip-LF, Eto-lip, and free etomidate dissolved in PBS (pH 7.4) or the commercial etomidate emulsion at a predetermined dose and was placed in its supine position. The next rat received an elevated dose (by a 0.1 log interval) if the former rat did not show LORR; otherwise, it received a reduced dose (by a 0.1 log interval). For each experiment, when there were six evaluable samples, the ED_50_ value was calculated based on the order of the events (i.e., success or failure to induce LORR in the rats) and the tested dose values corresponding to these events. The therapeutic indexes (i.e., the LD_50_/ED_50_ ratio) of Eto-lip-LF and Eto-lip were then calculated.

To investigate the onset time, action duration, and recovery time of Eto-lip-LF after anesthesia induction via a single injection, rats were randomized into four groups (n = 10). Each rat was intravenously injected with Eto-lip-LF, Eto-lip, free etomidate in PBS (pH 7.4), or the commercial etomidate emulsion (all at 2 mg etomidate/kg) and placed in its supine position. The time from the injection to LORR, the duration of LORR, and the time from the end of LORR to when the rat could stand on its own were recorded as the onset time, action duration, and recovery time, respectively.

To investigate the action duration and recovery time of Eto-lip-LF after anesthesia maintenance via continuous infusion, rats were randomized into four groups (n = 10). Each rat was intravenously injected with Eto-lip-LF, Eto-lip, free etomidate in PBS (pH 7.4), or the commercial etomidate emulsion (all at 2 mg etomidate/kg) to induce anesthesia, followed by continuous infusion of the same sample (all at 0.2 mg etomidate/kg/min) for 1 h using an LSP01-3A syringe pump (Baoding Dichuang Electronic Technology, Baoding, China). During the experiment, the status of each rat was monitored, and the action duration and recovery time were recorded right after the infusion finished.

### 2.11. Statistical Analysis

The data were analyzed using two-tailed Student’s *t*-tests for two groups and one-way ANOVA for multiple groups [46]. The data were reported as mean ± SD, unless otherwise indicated. Data differences with *p*-values < 0.05 were considered statistically significant.

## 3. Results and Discussion

### 3.1. Design and Preparation of Eto-Lip-LF

In this study, Eto-lip-LF was developed using EPC, Pluronic P123, and DSPE-PEG_2000_-LF as the loading materials. EPC, as a biocompatible and modifiable phospholipid, has been widely used to produce drug-loaded liposomes in pharmaceutical studies [47,48]. However, liposomes composed of EPC alone may not bring about satisfactory loading capacity, particle sizes, stability, and/or drug release profiles. Such problems also showed up in the encapsulation of etomidate during our pilot studies. Previously, we tried using EPC alone (100 mg) to encapsulate etomidate (10 mg), which resulted in large (average particle size > 140 nm) and unstable liposomes with fast drug release (drug release > 80% at 8 h under the same experimental conditions used in this study). Since hybrid drug delivery systems may make use of the advantages of different materials to obtain improved properties such as increased drug loading ability, more spherical shape and better homogeneity of the particles, desirable stability, and sustained drug release, other materials (e.g., Pluronic polymers, Tween 80, and chitosan) have been incorporated into EPC-based formulations [49,50]. In this study, it was hypothesized that the incorporation of lactoferrin together with Pluronic P123 in the EPC liposomes could achieve notably enhanced drug distribution in the brain and improved drug effects. Therefore, lactoferrin-modified Pluronic P123-incorporated liposomal etomidate Eto-lip-LF was produced. In addition, non-lactoferrin-containing liposomal etomidate Eto-lip as a specialized control for Eto-lip-LF was also prepared.

### 3.2. Characterizations and Stability Study

**Figure 2:**
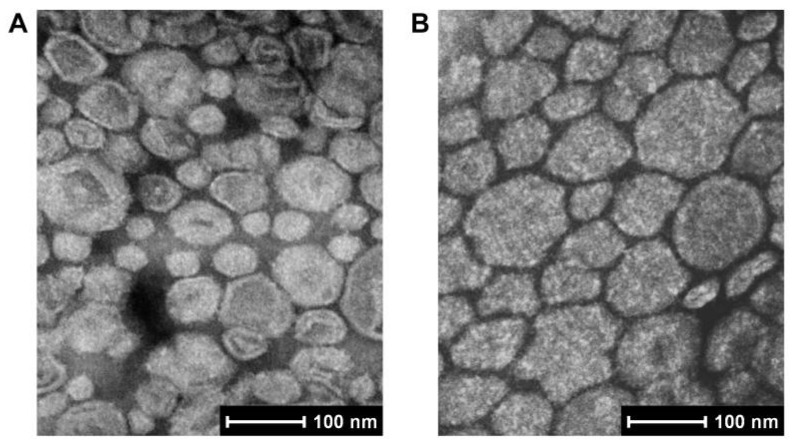
TEM images of (**A**) Eto-lip and (**B**) Eto-lip-LF. The liposomes were generally spherical, with shrinkage in some large ones. Compared with Eto-lip, Eto-lip-LF exhibited rougher surfaces, which probably resulted from more complex composition and particle structures.

The etomidate concentrations of both Eto-lip-LF and Eto-lip were approximately 2 mg/mL (Table 1). The average particle size was around 79 nm (PDI = 0.199 ± 0.016) for Eto-lip and around 115 nm (PDI = 0.298 ± 0.019) for Eto-lip-LF. The larger size and PDI of Eto-lip-LF were probably due to the incorporation of lactoferrin, which led to larger diameters and more complex structures of the liposomes. The TEM photos of Eto-lip-LF and Eto-lip are presented in Figure 2. In general, the liposomes were spherical, with shrinkage in some large ones. Nevertheless, the two formulations displayed varied appearances, probably due to different compositions of the liposomes. As shown in Table 1, both Eto-lip-LF and Eto-lip had very slight surface charges. The DLE of Eto-lip-LF was about 86%, close to that of Eto-lip (about 88%), suggesting good drug loading capacity of the liposomes without the incorporation of organic solvents or co-solvents. However, the DLC of Eto-lip-LF (about 5.1%) was significantly lower than that of Eto-lip (*p* < 0.001), which was due to the larger total amount of loading materials in Eto-lip-LF. It is noteworthy that although the DLE of the liposomes was not 100%, the small amount of drug that was not loaded by the liposomes could be dissolved in the PBS, which resulted in final drug concentrations of about 2 mg/mL.

**Figure 3:**
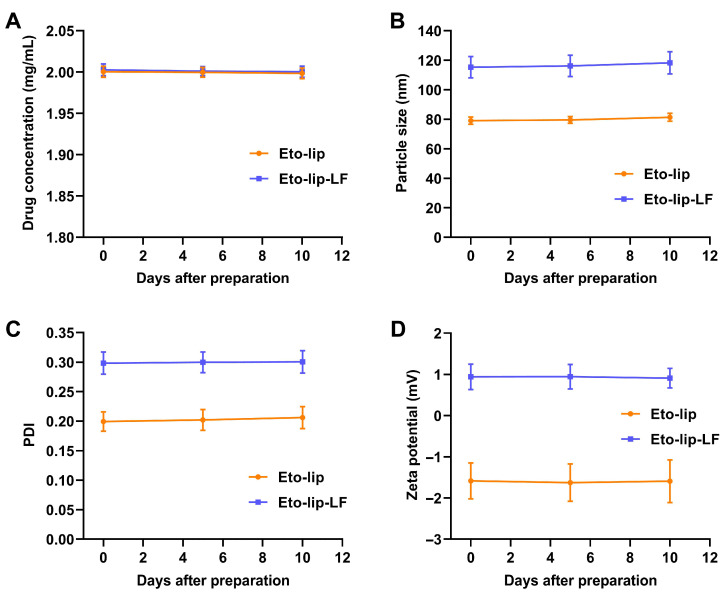
Changes in the (**A**) drug concentration, (**B**) particle size, (**C**) polydispersity index (PDI), and (**D**) zeta potential of Eto-lip-LF and Eto-lip were stored at room temperature for different days. Data are shown as mean ± SD (n = 3). The changes were negligible for both formulations on day 10, demonstrating the potential of Eto-lip-LF and Eto-lip to develop stable etomidate preparations for storage and transport at room temperature.

To study the stability of Eto-lip-LF, each liposome suspension was kept at room temperature (20–26 °C) for 10 days. During storage, all samples remained clear and colorless without observable precipitation and remained stable at concentrations of 2 mg/mL (Figure 3A). For the whole period of observation, the particle size, PDI, and zeta potential of both Eto-lip-LF and Eto-lip exhibited negligible changes (Figure 3B–D), indicating that the liposomal etomidate had the potential to develop as stable anesthetic agents for storage and transport at room temperature.

### 3.3. In Vitro Drug Release

**Figure 4:**
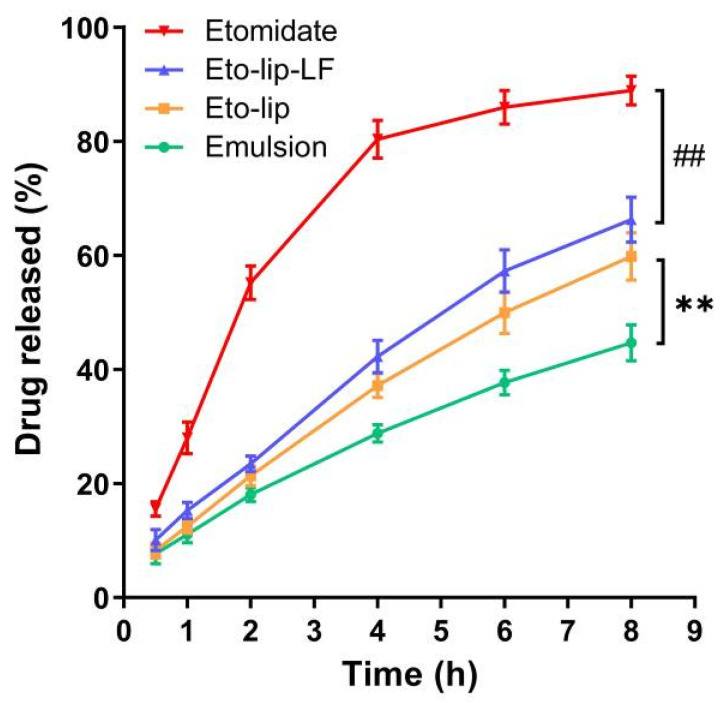
In vitro drug release of free etomidate dissolved in PBS (“Etomidate”), Eto-lip-LF, Eto-lip, and the commercial etomidate emulsion (“Emulsion”) at 37 °C. Data are shown as mean ± SD (n = 3). ^##^ *p* < 0.01 (Etomidate vs. Eto-lip-LF). ** *p* < 0.01 (Eto-lip vs. Emulsion).

Quick and complete release of etomidate from the nanocarriers is one of the essential factors to ensure rapid onset of general anesthesia and rapid recovery from anesthesia for better anesthetic effects and clinical safety [6]. Failure to induce general anesthesia and prolonged adverse effects may occur after sustained drug release. In this study, the free etomidate in PBS presented a rapid-release profile, with nearly 90% of the drug out of the dialysis membrane at 8 h (Figure 4). It should be noted that the solubility of etomidate in PBS (pH 7.4) is about 0.5 mg/mL at 37 °C [8]. For the free etomidate group, 10 mg etomidate was dissolved in 25 mL PBS (0.4 mg/mL) to ensure the dissolution of the drug. The commercial etomidate emulsion presented the slowest release profile, with only about 45% drug release at 8 h. Although the drug release of Eto-lip-LF and Eto-lip was also slower as compared with the free drug (*p* < 0.01 for Etomidate vs. Eto-lip-LF), it was still notably faster than that of the emulsion (*p* < 0.01 for Eto-lip vs. Emulsion). It was noteworthy that a considerable amount of soybean oil was used in the commercial emulsion to solubilize etomidate, which may not only cause problems for patients with obesity or hyperlipemia but also hinder the drug release due to the intense hydrophobic interactions between etomidate and soybean oil. The above result demonstrated the potential of Eto-lip-LF and Eto-lip to realize rapid induction of general anesthesia and rapid recovery for better clinical safety. Nevertheless, this study made only basic comparisons among the results of the four groups since the drug release profile of a formulation in vitro can be different from that of the formulation in vivo. A longer time range for drug release and investigation of in vivo drug release can be considered for a more comprehensive study in the future.

### 3.4. Hemolytic Activity

**Figure 5:**
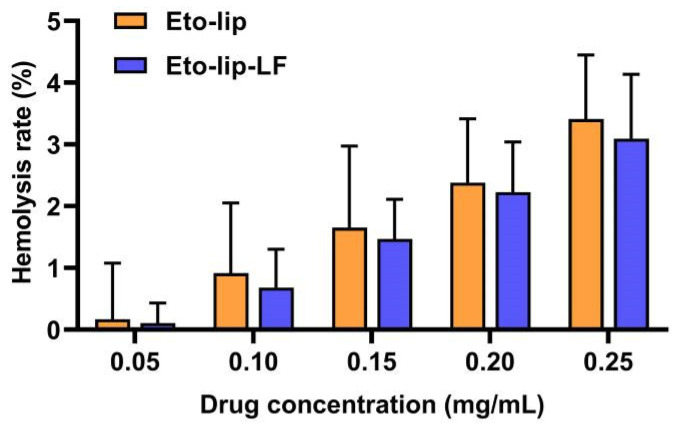
In vitro hemolytic activity of Eto-lip-LF and Eto-lip. Data are shown as mean ± SD (n = 3). The low hemolysis rate (<5%) of Eto-lip-LF and Eto-lip, even at the highest drug concentrations (0.25 mg etomidate/mL), indicated good hemocompatibility of both liposomal formulations.

The hemolytic activity of Eto-lip-LF and Eto-lip was studied to evaluate their blood compatibility, using the hemolysis rate as an indicator. Pure water and normal saline were used as the positive and negative controls, respectively. Generally, a hemolysis rate of <5% suggests acceptable hemocompatibility [51,52,53]. As shown in Figure 5, the liposomal etomidate caused hemolysis in a dose-dependent manner. However, for both formulations, low hemolytic activity was observed even at the highest drug concentration of 0.25 mg etomidate/mL. These results suggested the good hemocompatibility of Eto-lip-LF and Eto-lip as intravenous anesthetic agents.

### 3.5. Fluorescence Imaging

**Figure 6:**
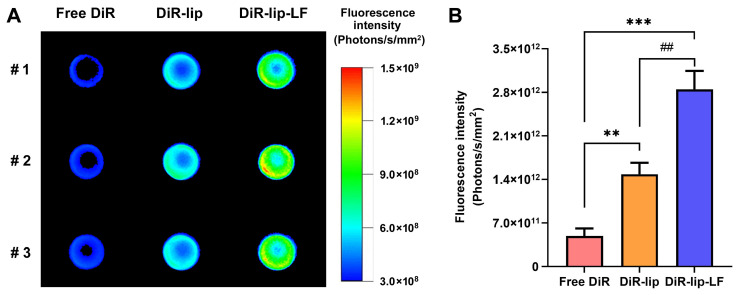
(**A**) Fluorescence imaging of homogenized brain tissues from the rats injected with free DiR dissolved in ethanol/PBS (1:4, *v*/*v*) mixed solvent, DiR-loaded liposome (DiR-lip), or DiR-loaded lactoferrin-modified liposome (DiR-lip-LF). (**B**) The fluorescence intensity of homogenized brain tissues from the rats. Data are shown as mean ± SD (n = 3). ** *p* < 0.01 (free DiR vs. DiR-lip). ^##^ *p* < 0.01 (DiR-lip vs. DiR-lip-LF). *** *p* < 0.001 (free DiR vs. DiR-lip-LF).

Near-infrared fluorescence imaging was conducted to investigate the brain-targeting ability of the liposomal nanoformulations. The rats were injected with 300 μL of free DiR or liposomal DiR (i.e., DiR-lip or DiR-lip-LF) with a dye concentration of 50 nmol/mL. Figure 6A shows the fluorescence images of homogenized brain tissues from the rats sacrificed 2 h after the injections. The free DiR group exhibited weak fluorescence intensity, which was due to the free distribution and rapid clearance of the dye in vivo [54]. Compared with the free DiR group, considerable fluorescence signals could be observed in the DiR-lip group. Nevertheless, the DiR-lip-LF group exhibited the strongest fluorescence intensity. The quantified fluorescence intensity of the three groups is presented in Figure 6B. Although both DiR-lip and DiR-lip-LF exhibited significantly higher fluorescence intensity than the free DiR group, DiR-lip-LF had the highest level of fluorescence signal, which was in accordance with the fluorescence image. The above results demonstrated the great potential of the lactoferrin-modified liposomal nanoformulations for brain-targeted drug delivery.

### 3.6. Acute Toxicity and MTD

**Figure 7:**
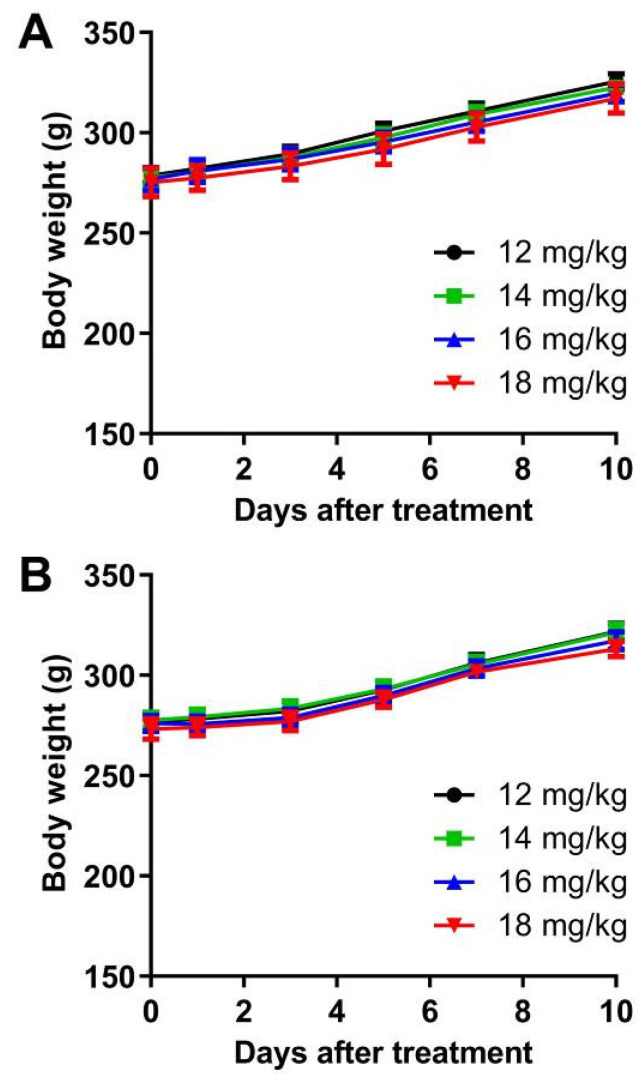
Body weight changes in the rats injected with (**A**) Eto-lip or (**B**) Eto-lip-LF at different dose levels. Data are shown as mean ± SD (n = 8 on day 0). No body weight loss was observed for the surviving animals at all dose levels (12–18 mg etomidate/kg).

The LD_50_ values of Eto-lip-LF and Eto-lip were 19.71 ± 0.90 mg etomidate/kg and 18.29 ± 1.57 mg etomidate/kg, respectively. This result was near our previously reported LD_50_ values of an etomidate-encapsulated micelle and the commercial etomidate emulsion (i.e., about 20 mg etomidate/kg), indicating the safety of the liposomal etomidate [8]. The MTD value was investigated as an indicator to determine the safe dose range for a single intravenous injection [55]. Four different dose levels (i.e., 12, 14, 16, and 18 mg etomidate/kg) near the LD_50_ values of the liposomal etomidate were tested in this experiment. The body weight and the status of the rats were monitored for 10 days post-injection. It was found that the rats could tolerate Eto-lip at the dose of 14 mg etomidate/kg and tolerate Eto-lip-LF at the dose of up to 16 mg etomidate/kg, with no death or other observable signs of toxicity (Table 2). The rats could tolerate a higher dose of etomidate with Eto-lip-LF than with Eto-lip, which may be due to the enhanced drug delivery of Eto-lip-LF to the brain. Enhanced drug delivery to the brain could increase the bioavailability of etomidate and reduce the effects (particularly adverse effects such as adrenal toxicity) of the drug on other organs/tissues, thus reducing the burden on the body caused by the drug. However, further study is needed to clarify the mechanisms for the toxicity of liposomal etomidate. Additionally, no body weight loss was found for the surviving rats at all dose levels, suggesting the safety of the liposomal etomidate for intravenous injection (Figure 7).

### 3.7. In Vivo Anesthetic Effects

**Figure 8:**
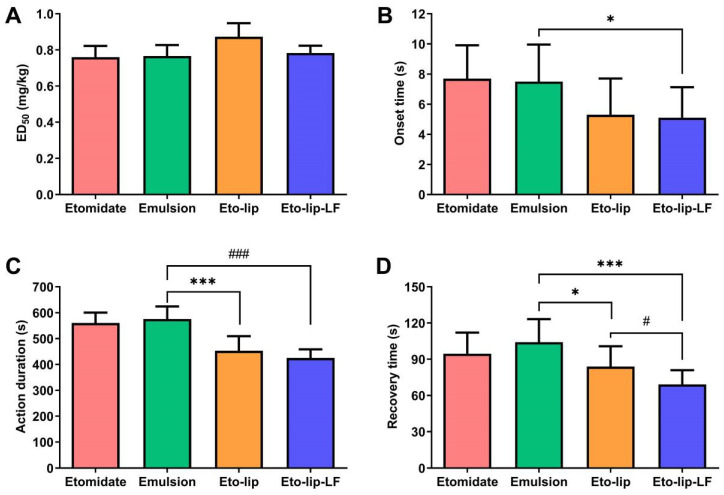
(**A**) ED_50_ values of free etomidate dissolved in PBS (“Etomidate”), the commercial etomidate emulsion (“Emulsion”), Eto-lip, and Eto-lip-LF in rats. Data are shown as mean ± SD (n = 3). The average ED_50_ of Eto-lip was a little higher than those of the other three groups, but the differences were not statistically significant (*p* > 0.05). (**B**) The onset time, (**C**) action duration, and (**D**) recovery time of the four groups after anesthesia induction via a single injection at 2 mg etomidate/kg. Data are shown as mean ± SD (n = 10). Eto-lip-LF showed a significantly faster onset (* *p* < 0.05) than the commercial etomidate emulsion. Both Eto-lip and Eto-lip-LF showed shorter action duration (*** *p* < 0.001 for Eto-lip vs. Emulsion, ^###^ *p* < 0.001 for Eto-lip-LF vs. Emulsion) and faster recovery (* *p* < 0.05 for Eto-lip vs. Emulsion, *** *p* < 0.001 for Eto-lip-LF vs. Emulsion) than the commercial etomidate emulsion. Moreover, Eto-lip-LF showed even faster recovery as compared with Eto-lip (^#^ *p* < 0.05).

The anesthetic effects of Eto-lip-LF in vivo were evaluated based on the LORR in rats. LORR is a useful and tractable indicator to determine loss of consciousness and has been widely used in the studies of general anesthetics [56,57,58,59]. To determine the proper dose for anesthesia induction, the ED_50_ value of Eto-lip-LF was investigated in the first place. As shown in Figure 8A, the ED_50_ of the free etomidate group was about 0.76 mg/kg, which was consistent with the result of early research [5]. The ED_50_ value was 0.77 ± 0.06 mg etomidate/kg for the commercial emulsion and 0.78 ± 0.04 mg etomidate/kg for Eto-lip-LF, close to that of the free drug. Although Eto-lip had a slightly larger ED_50_ (0.87 ± 0.08 mg etomidate/kg), the differences among all four groups were not statistically significant (*p* > 0.05). Based on the LD_50_ and ED_50_ values, the therapeutic index was about 25.3 for Eto-lip-LF and about 21.0 for Eto-lip, higher than that of many other clinically used general anesthetics, indicating the good safety of the liposomal etomidate [60].

The onset time, action duration, and recovery time of Eto-lip-LF after anesthesia induction via a single injection were investigated at 2 mg etomidate/kg, which was a little higher than twice as much as the ED_50_ values. It was found that the onset time of the commercial emulsion (7.5 ± 2.5 s) was very close to that of the free drug (7.7 ± 2.2 s), as shown in Figure 8B. Both Eto-lip and Eto-lip-LF took effect more rapidly than the commercial emulsion, and the difference between Eto-lip-LF and the emulsion was statistically significant (*p* < 0.05), which was probably due to the brain-targeted components of the liposomes. Also, both Eto-lip and Eto-lip-LF had significantly shorter action durations (*p* < 0.001) than the commercial emulsion (Figure 8C). As shown in Figure 8D, the commercial emulsion had a slightly slower recovery (104.1 ± 19.1 s) than the free drug (94.6 ± 17.5 s). The recovery time of Eto-lip was 83.9 ± 16.9 s, shorter than that of the emulsion (*p* < 0.05). Moreover, Eto-lip-LF showed even faster recovery (69.2 ± 11.7 s) as compared with Eto-lip (*p* < 0.05). It is noteworthy that free etomidate could reversibly bind the plasma proteins (e.g., albumin), which may extend its residence time in the blood circulation, consequently reducing the amount and speed of etomidate entering the brain and also slowing down the drug metabolism [5,61]. In addition, for the emulsion, the slower drug release (as discussed above) may have further prolonged the action duration and recovery time of the etomidate. Therefore, the rapid onset and faster recovery of Eto-lip-LF and Eto-lip were probably achieved based on the protection of etomidate by the liposomes and the enhanced drug delivery to the brain.

**Figure 9:**
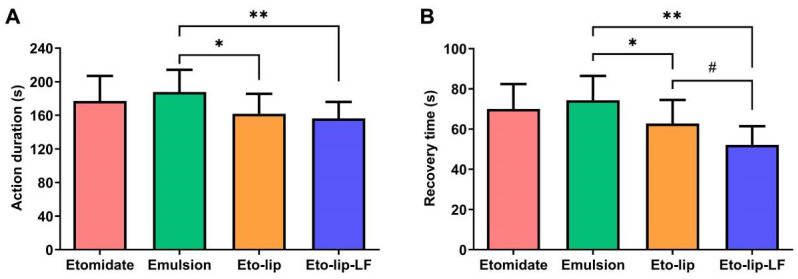
(**A**) The action duration and (**B**) recovery time of free etomidate dissolved in PBS (“Etomidate”), the commercial etomidate emulsion (“Emulsion”), Eto-lip, and Eto-lip-LF after anesthesia induction via a single injection at 2 mg etomidate/kg and anesthesia maintenance via continuous infusion at 0.2 mg etomidate/kg/min for 1 h. Data are shown as mean ± SD (n = 10). Both Eto-lip and Eto-lip-LF showed shorter action duration (* *p* < 0.05 for Eto-lip vs. Emulsion, ** *p* < 0.01 for Eto-lip-LF vs. Emulsion) and faster recovery (* *p* < 0.05 for Eto-lip vs. Emulsion, ** *p* < 0.01 for Eto-lip-LF vs. Emulsion) than the commercial etomidate emulsion. Moreover, Eto-lip-LF showed faster recovery as compared with Eto-lip (^#^ *p* < 0.05), which indicated its potential for clinical safety.

Like anesthesia induction, anesthesia maintenance is also a basic need for clinical practice, particularly for surgical operations. However, so far, there have been very few reports on the anesthesia maintenance of etomidate and its preparations. In this study, the action duration and recovery time of Eto-lip-LF after anesthesia maintenance for 1 h via continuous infusion at 0.2 mg etomidate/kg/min were investigated. Throughout this experiment, no deaths or other observable signs of toxicity occurred in the animals. As shown in Figure 9, the action duration and recovery time of the free drug group were about 177.1 s and 70.1 s, respectively. Compared with the free drug, the commercial emulsion showed a slightly longer action duration (187.9 ± 26.3 s) and recovery time (74.4 ± 12.1 s), but the differences were not statistically significant (*p* > 0.05). The action duration and recovery time of Eto-lip were about 161.8 s and 62.8 s, respectively, significantly shorter than those of the commercial emulsion (*p* < 0.05). Nevertheless, Eto-lip-LF had an action duration of 156.4 ± 19.6 s and a recovery time of 52.2 ± 9.3 s, even shorter than those of Eto-lip (*p* < 0.05 for the recovery time). These results further suggested the safety of Eto-lip-LF for general anesthesia.

In summary, the results of this study demonstrated the high drug loading efficiency and stability of the liposomal formulations without the incorporation of organic solvents or co-solvents. Eto-lip-LF exhibited excellent hemocompatibility in vitro and good safety in vivo. Moreover, the animal studies supported our hypothesis that the incorporation of lactoferrin together with Pluronic P123 in the liposomal etomidate could achieve notably enhanced drug distribution in the brain and improved general anesthetic effects. These results suggested that this novel etomidate preparation was a promising anesthetic delivery system that warranted further development for clinical applications. It is noteworthy that although the experiments and their sample sizes in this initial study allowed for basic statistical analyses for comparisons, the sample sizes of some experiments, such as fluorescence imaging and those about in vivo drug effects, may need to be properly increased for a more comprehensive study of Eto-lip-LF in the future.

## 4. Conclusions

In this study, liposomal etomidate (Eto-lip-LF) as a novel nanoformulation for general anesthesia was successfully developed. This lactoferrin-modified liposomal etomidate exhibited good drug loading capacity without the incorporation of organic solvents or co-solvents and achieved a high DLE of about 86%. Our stability studies demonstrated the potential of Eto-lip-LF to develop as a stable etomidate preparation for storage and transport at room temperature. Compared with the non-lactoferrin-containing liposomes, the lactoferrin-modified liposomes exhibited notably enhanced brain-targeting ability, which we believe to have resulted from the binding of transferrin with the transferrin and lactoferrin receptors highly distributed in the brain. Eto-lip-LF showed good hemocompatibility and a wide safe dose range for intravenous injection in rats, with an LD_50_ of 19.71 mg etomidate/kg and an MTD of 16 mg etomidate/kg. The therapeutic index of Eto-lip-LF was about 25.3, higher than that of many other general anesthetics, further indicating the safety of this etomidate preparation. Moreover, as compared with the commercial etomidate emulsion, Eto-lip-LF could better achieve rapid onset of general anesthesia and rapid recovery from general anesthesia, which was probably achieved based on enhanced drug delivery to the brain. Taken together, lactoferrin-modified liposomal etomidate was promising to become an alternative preparation for clinical general anesthesia. Nevertheless, the biodistribution of Eto-lip-LF and the effects of this formulation on drug delivery in vivo need more systematic investigation. In addition, detailed studies on the toxic side effects of Eto-lip-LF and the related mechanisms are planned.

## Figures and Tables

**Table 1:** The characterization results of Eto-lip and Eto-lip-LF. Data are presented as mean ± SD (n = 3). *** *p* < 0.001 as compared with Eto-lip.

Investigated Items	Eto-Lip	Eto-Lip-LF
Concentration (mg/mL)	2.001 ± 0.007	2.003 ± 0.007
Particle size (nm)	79.1 ± 2.5	115.2 ± 7.3
PDI	0.199 ± 0.016	0.298 ± 0.019
Zeta potential (mV)	−1.59 ± 0.44	0.94 ± 0.31
DLE (%)	88.29 ± 1.22	85.95 ± 1.15
DLC (%)	8.11 ± 0.10	5.10 ± 0.06 ***

**Table 2:** Maximum tolerated dose (MTD) of Eto-lip and Eto-lip-LF.

Sample	Dose (mg Etomidate/kg)	Rat Death	Weight Change on Day 10 (%)
Eto-lip	12	0/8	+16.87
	14	0/8	+16.16
	16	2/8	+15.41
	18	4/8	+15.17
Eto-lip-LF	12	0/8	+16.26
	14	0/8	+15.71
	16	0/8	+14.95
	18	3/8	+14.64

## Data Availability

Data are available upon reasonable request from the corresponding author.
